# Nanoengineered Platforms to Guide Pluripotent Stem Cell Fate

**DOI:** 10.4172/2157-7439.1000217

**Published:** 2014-08-12

**Authors:** Katy Rutledge, Ehsan Jabbarzadeh

**Affiliations:** 1 Department of Chemical Engineering, University of South Carolina, Columbia, SC, 29208, USA; 2 Biomedical Engineering Program, University of South Carolina, Columbia, SC, 29208, USA; 3 Department of Orthopaedic Surgery, University of South Carolina School of Medicine, Columbia, SC, 29209, USA

**Keywords:** Embryonic stem cells, Induced pluripotent stem cells, Stem cell microenvironment, Nanotechnology, Physical signals, Cell shape, Surface topography

## Abstract

Tissue engineering utilizes cells, signaling molecules, and scaffolds towards creating functional tissue to repair damaged organs. Pluripotent stem cells (PSCs) are a promising cell source due to their ability to self-renewal indefinitely and their potential to differentiate into almost any cell type. Great strides have been taken to parse the physiological mechanisms by which PSCs respond to their microenvironment and commit to a specific lineage. The combination of physical cues and chemical factors is thought to have the most profound influence on stem cell behavior, therefore a major focus of tissue engineering strategies is scaffold design to incorporate these signals. One overlooked component of the *in vivo* microenvironment researchers attempt to recapitulate with three dimensional (3D) substrates is the nanoarchitecture formed by the fibrillar network of extracellular matrix (ECM) proteins. These nanoscale features have the ability to impact cell adhesion, migration, proliferation, and lineage commitment. Significant advances have been made in deciphering how these nanoscale cues interact with stem cells to determine phenotype, but much is still unknown as to how the interplay between physical and chemical signals regulate *in vitro* and *in vivo* cellular fate. This review dives deeper to investigate nanoscale platforms for engineering tissue, as well use the use of these nanotechnologies to drive pluripotent stem cell lineage determination.

## Introduction

### Tissue engineering

In recent years, tissue engineering has emerged as a potential method for treating numerous diseases and regenerating damaged cells. By applying engineering approaches to knowledge of biological systems, a tissue engineered substitute can be generated to restore, replace, or maintain partial if not entire organ function [1]. The key to tissue engineering strategies is coordinating cell behavior with specific growth factors and biomaterials in order to regenerate functional tissues, however this is difficult to experimentally control.

Towards this effort of fully directing cell behavior, various biomaterials as well as different cell types and signaling molecules have been investigated. Recent studies have examined the ability of mechanical signals to influence stem cell lineage commitment since cells in the body reside in different tissue niches with variable mechanical properties that effect cellular function. From this information, biomaterials have been designed to harness tissue-specific mechanical properties to guide stem cells to the targeted cell type. Specifically, nanoscale platforms are utilized since they offer a unique ability to mimic the physical cues that cells receive from their microenvironment. Researchers have developed materials with tunable matrix elasticities, nanotopographies, and nanoscale patterns that have the ability to manipulate cell phenotype. This review explores current developments in the use of nanotechnology to drive cell function determination. The following section briefly describes common cell types used in tissue engineering applications, then discusses the interactions between cells and different biomaterials with nanoscale features.

### Cell sources

Major sources of cells for tissue engineering include adult stem cells, progenitor cells, embryonic stem cells (hESCs), and induced pluripotent stem cells (iPSCs). Adult stem cells, or mesenchymal stem cells (hMSCs), are multipotent and generally derived from adipose tissue (AD-hMSCs) or the bone marrow (BM-hMSCs). When exposed to the correct chemical signals, AD-hMSCs can differentiate towards osteogenic, chondrogenic, adipogenic, myogenic, and hepatic lineages, as well as become endothelial cells [2-5]. Bone marrow-derived hMSCs are a type of adult stem cell that can differentiate to bone, cartilage, muscle, ligament, tendon, adipose, and stroma lineages [6-9]. Mesenchymal stem cells are useful in numerous applications, however are subject to senescence and cannot indefinitely proliferate [10]. Furthermore, when hMSCs are removed from their native stem cell microenvironment, they lose differentiation potential at a rapid pace [11].

Progenitor cells are lineage-committed and maintain the tissue in which they reside [10]. These progenitor cells have been discovered in muscle tissue [12], cartilage [13], bone [13,14], tissue in the central nervous system [15], in the bulge of the hair follicle [16], as well as many other locations. Like hMSCs, progenitor cells have a finite limit to proliferation and follow Hayfick's limit of 50-70 population doublings [10,17]. Both hMSCs and progenitor cells have the advantage of patient specific treatment, however their narrow differentiation capabilities and limited proliferation potential present severe drawbacks.

### Advantages of pluripotent stem cells

hESCs and iPSCs overcome limitations of hMSCs and progenitor cells by offering the ability to self-renew indefinitely as well as differentiate into any cell type of the body [18]. Embryonic stem cells are derived from the inner cell mass of a blastocyst during the pre implantation stage [19-21]. hESCs are typically grown in colonies and maintained in feeder-dependent or feeder-free conditions to keep cells in an undifferentiated state. In traditional two-dimensional (2D) cell culture, hESC colonies are grown on a feeder layer of mitotically inactivated mouse embryonic fibroblasts. To avoid using cell types outside of hESCs for differentiation studies, 2D well plates can be coated with matrigel to allow for hESC attachment without the feeder layer, on which cells are able to double 130 times before losing pluripotency [22]. This is crucial in conducting successful differentiation studies. Undifferentiated colonies can be transformed into embryoid bodies (EBs) that are useful in differentiation experiments since they mimic the natural developmental morphology during gastrulation and the *in vivo* germ layer establishment [23]. While the three-dimensionality of EBs is ideal, a drawback of this culture method is the inability to generate a large number of cells. The disparity between *in vivo* and *in vitro* tissue development from hESCs can be overcome by engineering a three-dimensional (3D) microenvironment from which the undifferentiated cells can receive cues and thus differentiate towards specific lineages.

iPSCs were first created by introducing four pluripotency transcription factors (Oct3/4, Sox2. C-Myc, and Klf4) to a mouse fibroblast cell, after which the fibroblast exhibited properties of undifferentiated hESCs [24]. These stem cells have the ability to differentiate into cells of all three germ layers, can self-renew, and proliferate indefinitely [25,26]. Unlike hESCs, iPSCs offer the opportunity for patient specific treatment since somatic cells can be taken from the target host, reprogrammed through the addition of transcription factors, cultured to increase cell number, differentiated towards the desired lineage, and finally implanted back into the patient. Since the genetic material in the implanted cells is the same as in the host's cells, the risk of immunorejection is low.

One barrier for using hESCs and iPSCs in regenerative medicine is that teratoma formation in implanted tissue can occur when cells have not fully and uniformly differentiated into the target tissue [27,28]. Therefore, it is extremely important to develop a direct approach to exclusively generating desired cells and avoiding spontaneous teratoma formation.

## Microenvironmental Influence on Pluripotent Stem Cell Phenotype

Although hESCs and iPSCs are promising cell sources for tissue engineering applications and invaluable tools for studying developmental biology, there are still many fundamental aspects of PSC biology that are unknown. Specifically, researchers are striving to understand and deconstruct the mechanisms by which the microenvironment effects lineage determination, as well as cell phenotype and function.

The native microenvironment is composed of the extracellular matrix (ECM), which is a network of proteins that provides physical and chemical cues determining cell behavior [29-32]. Cell biologists have analyzed numerous cytokines and soluble factors responsible for stem cell regulation, however, recent studies indicate that these soluble factors work in conjunction with the insoluble components present in the ECM such as adhesive, mechanical, and topographical cues [33-37]. Specifically, insoluble factors are made up of collagens, non-collagenous glycoproteins (laminin, elastin, fibronectin), and hydrophilic proteoglycans [38]. Stem cells can detect and respond to signals simultaneously presented in the microenvironment; cell mechanotransduction machinery converts these soluble and insoluble cues to signal upregulation of various genes and subsequent lineage commitment [37].

**Figure 1:**
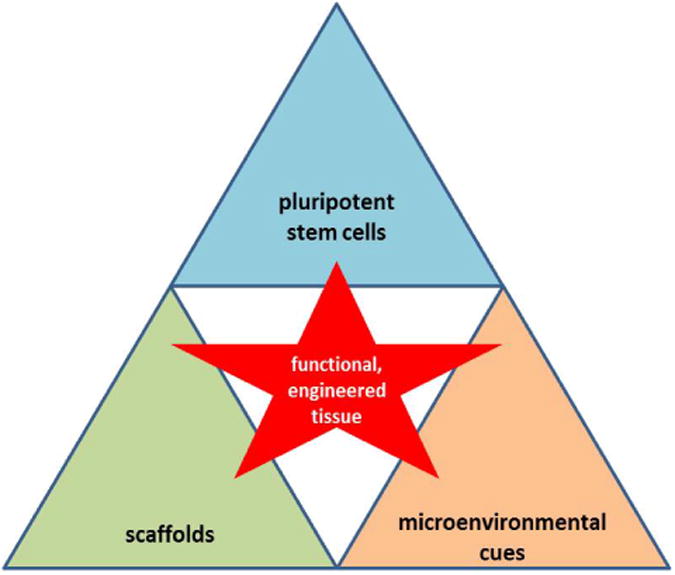
Tissue engineering coordinates the interplay of cells, biomaterials, and signals to create the desired functional tissue. This review investigates pluripotent stem cells and how nanotechnology-incorporated scaffolds can provide physical cues to direct cellular fate.

Past biomaterial design has focused on microscale technologies to drive stem cell lineage commitment, but the *in vivo* tissue structure provides cues to cells at a nanoscale. Furthermore, cells tend to respond to microscale fiber scaffolds the same way that they do when cultured on a 2D polystyrene cell culture plate. Cell morphology becomes fat, which causes a lopsided attachment of focal adhesions [38]. Therefore, providing signals at the microscale level might be physiologically inconsistent for directing stem cell differentiation [39], and there is a need to engineer functional nanoscale microenvironments for tissue engineering applications. The field of nanotechnology in relation to tissue engineering involves designing novel materials with at least one dimension between 1-100 nm to use as scaffolds for influencing cell behavior [40]. The following section will discuss different techniques for creating biomaterials with nanoarchitectural features (Figure 1).

### Nanoscale platforms

The *in vivo* microenvironment is composed of channels, pores, and ridges that provide physical cues to cells at a nano level [39]. Knowledge of how these factors influence stem cell behavior is necessary to effectively design scaffolds that differentiate stem cells to the desired lineage. To analyze the impact of nanofeatures on cell behavior, engineers and scientists have combined principles of chemistry, physics, material science, and biology to create specialized substrates. Fabrication techniques such as soft lithography, deposition of nanostructures, microfluidics, and electrospinning all create ways for researchers to manipulate topography [41-43]. These platforms have been used to determine specific cues that regulate stem cell function. In this section, current nanotechnology and material approaches are introduced, and applications of these platforms will be addressed later on in the review.

### Electrospinning

This technique can form a network of polymer fibers down to the size of 10 nm [44]. To generate electrospun scaffolds, a voltage is applied to a polymer solution, the charged solution is ejected though a needle, and electric forces stretch the polymer jet so that fibers with submicroscale diameters form on the grounded collector surface [45]. Since the fiber diameters are much smaller than cellular surface area, this platform allows cells to organize around the fibers [46] and attach with a spread morphology with numerous focal adhesions [47]. Another advantage of this technique is the ability to create electrospun scaffolds from synthetic as well as natural polymers [45]. However, one challenge with this fabrication method is that cells cannot migrate throughout the scaffold due to pore sizes being smaller than that of a cell [38]. Recent progress has overcome this limitation by using self-assembly of nanofibers around the cells [48]. Knowledge of protein self-assembly and optimization of noncovalent intermolecular interactions produced this revolutionary approach to forming the nanofibrillar architecture around cells without damaging them [49,50]. This technique allows the scientist to spatially and mechanically organize cells, which is critical to tissue engineering strategies since cells in the body are arranged in specific patterns that form tissues and organs [38]. This fabrication process is able to create substrates that mimic grooves, ridges, and the fibrillar ECM structure, and recent advancements with assembling the scaffold material around target cells has overcome the previous inability of cells to infiltrate the scaffold.

### Soft lithography

The general method of soft lithography uses elastomeric stamps to print nanoscale polymers on a surface [51-54]. Patterned polymers can range from 30 nm to several microns [55]. This is a useful technique because the engineer has full control over spatial distribution of polymer molecules placed on the substrate, which subsequently determines cell spreading and shape [56-58]. Soft lithography is an invaluable tool because it creates a platform on which researchers can isolate and control mechanical cues exposed to single cells [59] and also pairs or triplets of cells [60]. This technique is very useful in deciphering cellular reactions to individual physical cues since the polymers can be easily manipulated to express specific mechanical characteristics. However, a limitation with this platform is that soft lithography is only able to provide a narrow range of ECM signals for the cell to receive, which is inconsistent with the many microenvironmental cues provided to cells *in vivo*.

### Hydrogels

Hydrogels are a popular tissue engineering scaffold with proven success in medicine and biological research due to their tunable tissue-like properties [61-65]. The goal of hydrogel design is to mimic natural ECM, which is accomplished by crosslinking polymers. The intricate linking of these hydrophilic molecules forms a network with tissue-like viscoelastic mechanical properties, as well as similar interstitial flow to the *in vivo* microenvironment. Similar diffusive transport also occurs in hydrogel cell culture platforms, and hydrogels can be designed to incorporate cell adhesion ligands and other biologically relevant components [38]. Although hydrogels are an extremely moldable substrate and offer numerous advantages as scaffolds for tissue engineering, they have low mechanical strength, they are difficult to sterilize, and loading drugs and cells in the matrix before crosslinking the material is difficult [66,67]. Further optimization studies are warranted to overcome these barriers.

### Carbon nanotubes

Carbon nanotubes (CNTs) possess ideal qualities for tissue regeneration strategies such as tunable chemical and mechanical properties, electrical conductivity, cytocompatibility, and nanoscale dimensions that serve as topographical cues [68]. Furthermore, CNTs have numerous applications for directing cell behavior such as drug delivery, gene modifications, and incorporation in the *in vitro* 3D cell microenvironment to add roughness [69-71]. When placed in fetal bovine serum (FBS), proteins readily adsorb to the surface of CNTs subsequently promoting cell attachment [72]. Several studies have demonstrated the potential of CNTs for bone tissue engineering applications [73-76], myoblastic cell attachment and growth [72] as well as neuronal cell proliferation [77], but little is known regarding the effect of CNTs on stem cell fate. In order to hypothesize how CNTs could influence pluripotent stem cell behavior, an analysis of studies conducted on the effect of CNTs on other cell types must be done, with further scrutiny on how individual characteristics of CNTs control the cellular response. Towards this goal, a recent study has shown that the mechanical properties of CNTs promote differentiation of MC3T3-E1 osteoblasts towards osteogenic lineage [73]. A study isolating the conductivity attribute of CNTs demonstrated that multiwall carbon nanotube (MWNT)-incorporated hydrogels increased cell proliferation of myocytes as well as fostered the growth of multinucleated cells with higher actin filament interactions as compared to the control groups [78]. CNTs can be functionalized to exhibit varying chemical properties that influence cell phenotype, shown in a study investigating single wall carbon nanotube (SWNT) conjugation with poly(*m*-aminobenzene sulfonic acid) and polyethylene glycol in which neurons exposed to more positively charged groups exhibited greater neurite length and had additional growth cones [79]. Since CNTs exhibit numerous traits that have the potential to impact cell lineage commitment, there is a need to delineate the effects of each specific CNT characteristic on hPSC behavior.

### Microfluidics

This technique allows for precise regulation of fluid flow and microenvironmental geometry, usually in the form of channels with similar dimensions to that of the cell type under investigation. Volumes can easily be controlled to levels of 10^-18^ liters [80], and the flow rates are manipulated so that shear stress in the *in vitro* microenvironment is optimized. Microfluidic platforms have been used extensively to study cell biology, specifically cellular adhesion forces [81], the cytoskeleton [82], and the culture of embryos [83-85]. This platform is useful in determining the influence of shear stress on individual cells, as well as mimicking the effects of capillary and interstitial flow, but the scale of this technique is not practical for larger magnitude tissue regeneration studies.

## Biophysical Signals for Differentiating Pluripotent Stem Cells

As described in the previous section, several methods for tuning biophysical signals for influencing stem cell behavior have been explored. Nanoscale signals such as shear stress, strain, material elasticity, topographical variation, and cell shape all affect cellular function and lineage specification. This section discusses how each of these factors govern PSC behavior; specifically cell adhesion, proliferation, alignment, and differentiation.

### Mechanical cues

Cells experience mechanical cues such as stress and strain in the *in vivo* microenvironment. Muscle contraction and relaxation, bone compression and decompression, cell migration, fluid flow, and tissue regeneration all cause variations of mechanical forces in the body. The ECM also has a range of elastic moduli that generate physical stimuli for attached cells through focal adhesions. These mechanical cues are transduced through focal adhesion kinase (FAK) and Src family signaling [37]. Furthermore, integrins are activated by stress, strain, and differing elastic moduli. This in turn increases focal adhesion strength and upregulates integrin mediated signaling throughout the cell. The biochemical pathways that are activated as a result of physical stimuli are part of a positive-feedback loop which further activates actomyosin cytoskeleton tension and increases focal adhesion strength [37,86,87]. As a result, researchers have explored how varying signals alter hESC behavior *in vitro* with hopes of determining what physical forces, separately or in combination, control lineage commitment.

#### Cyclic strain and stress

The effect of mechanical cues on cell function has been studied for many cell types, and has established general knowledge regarding cell behavioral responses [36,88-91]. Recent studies have made great strides in determining the impact physical stimuli have on hESCs. For example, the effect of cyclic strains on hESCs inhibited differentiation and increased self-renewal [92]. This was caused by the upregulation of TGFβ1, Activin A, and nodal which initiates the phosphorylation of Smad 2/3 [93]. Another study showed that cyclic stress through integrin-mediated adhesions induces spreading of mouse ESCs and decreased the expression of pluripotency marker Oct3/4 [94].

#### Shear stress

Shear flow has also been investigated as a mechanical cue for PSCs since it is a dynamic stress found *in vivo*, most commonly exerted on cells in the circulatory system [95]. Mouse ESCs placed in a microfluidic chamber demonstrated that a higher flow rate of 1.1 μL/min produced larger, round colonies as compared to slower rates of 0.001 μL/min and 0.019 μL/min [96]. This rounded phenotype indicates decreased cytoskeletal tension. When ESCs are subjected to highly controlled shear flow they differentiate into endothelial or specialized cardiovascular cells [97,98]. Furthermore, ESCs exposed to culture conditions with shear stress express greater levels of endothelial cell proteins CD31 and Runx1, and cells formed hematopoietic colonies [99].

#### Material properties

A recent study demonstrated that matrix elasticities of 1 kPA, 8 kPa, and 25 kPa lead hMSCs respectively towards neurogenic, myogenic, and osteogenic lineage [91]. This discovery, along with the known fact that matrix mechanics are a definitive factor in tissue morphogenesis and cell function [100-102], influenced researchers to investigate the response of ESCs to material properties.

ESCs are generally cultured on stiff 2D cell culture plates. Studies have shown that cell traction and colony stiffness increase when ESCs are grown on traditional rigid substrates, which also correlates with the downregulation of Oct3/4 in mouse ESCs [94,103]. Cells grown on soft polyacrylamide gels with a stiffness of 0.6 kPA formed round, compact colonies that had high Oct3/4, Nanog, and Alkaline Phosphatase expression compared to the polystyrene plates with stiffness of approximately 4 MPa [103]. This study demonstrated that soft materials cause cells to exhibit low traction forces and colony stiffness, and as a result, self-renewal and pluripotency of ESCs is maintained.

### Cell shape

Stem cell shape regulates physiology, controls proliferation, and ultimately governs lineage specification [104, 105]. Cells have particular shapes that optimize carrying out specific cellular functions: neurons have long bodies to efficiently deliver signals that can span the entire length of the human figure, where adipocytes are spherical to store lipids [37]. From a developmental point of view, signals from the stem cell niche induce conformational changes which then influence tissue structure and purpose [106-108].

One of the first experiments demonstrating the impact of cell size on behavior used 20 μm^2^ and 75 μm^2^ fibronectin islands to show that size directly controls apoptosis and proliferation, respectively [109]. Furthermore, studies have shown that restricting hESC colony size regulates differentiation, with smaller cell groupings favoring endoderm commitment over ectoderm [110]. Patterning adhesive ligands to control hESC colony size determined large colonies with a high cell density microenvironment promote pluripotency, controlled through a BMP-mediated Smad1 gradient. This gradient forms due to the interaction of hESCs and hESC-derived extraembryonic endoderm [111]. These findings are thought to occur due to cell-cell contact, varying mechanical stresses throughout the body of cells, and soluble factor gradients.

### Topographical cues

Topography plays a key role in cell maintenance and function. Nanoscale architecture has grooves, ridges, pits, and pores *in vivo*; for example proteins in the ECM are generally arranged in a fibrous manner with these topographical properties. These fibrillar networks are approximately 10-100 nanometers but can be several microns [112,113], and the bone marrow contains numerous nanoscale pores that provide additional cues for stem cells [95]. Nanotopography is important because cells receive signals through specific binding sites that integrins recognize, and integrin signaling is controlled through nanoscale ECM-cell interactions [39]. Surface features as small as 10 nm have the ability to influence cell adhesion [114]. When cells bind to integrins, tyrosine kinase and phosphatase signaling is activated, and both are important for cell fate and gene expression [37]. Trough these biophysical cues, stem cell adhesion and cytoskeleton organization are regulated, thus cell decisions regarding proliferation, migration, elongation, cell alignment, polarization and differentiation are impacted [112,115-122]. Studies using MSCs determined that the nanoscale topography potentially acts through spatial control of ligands and regulatory factors, and the interplay between physical and biochemical cues determine cell morphology and phenotype [95]. This, among other principles discovered by examining hMSC response to alterations in topography, can be applied to hPSCs.

Topography is a powerful tool since, not only is cytoskeleton tension altered like in cell shape experiments, but also entire molecular arrangement and dynamic organization of cellular adhesion mechanisms are affected [37]. Polymethylglutarimide (PMGI) nanofibers were used as scaffolds to maintain mouse ESC stemness and it was concluded that fiber density and structure were important factors in retaining pluripotency. This study also found that mouse ESCs had the ability to differentiate into all three germ layers on this substrate [123]. Studies observing the response of hESCs to nanotopography have used fibronectin coated poly(di-methyl siloxane) (PDMS) substrates with 600 nm ridges, 600 nm spacing, and 600 +/- 150 nm height. Single cells were placed on the surface for 24 and 48 hours, and it was determined that the nanotopographic surfaces increased cell alignment and elongation, but decreased projected cell area and proliferation [124]. An experiment utilizing polyamide nanofibrillar surfaces covalently linked to FGF-2 found that this substrate enhanced hESC proliferation [125]. The use of fibrillar nanoarchitecture in scaffolds has the potential to spatially align and organize cells while retaining pluripotency, however cell proliferation capabilities depends on the ridge size and surface chemistry of the scaffold and must be further optimized to sustain hPSC growth.

**Figure 2:**
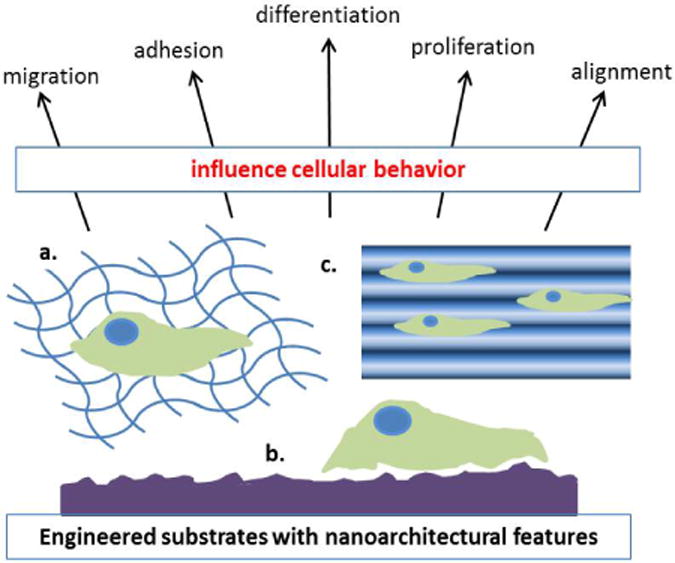
Biomaterials with (a) fibrous architecture, (b) surface roughness and varying nanotopographical features, and (c) nano grooves/ridges provide cues to cells. These microenvironmental signals, along with other mechanical cues mentioned in this review, have the ability to influence cell migration, adhesion, differentiation, proliferation, and alignment.

Another study used UV-assisted capillary force lithography to create 350 nm ridge/groove pattern arrays, then demonstrated the ability of the surface topography to direct hESCs towards neuronal lineage in the absence of differentiation-inducing soluble factors [126]. Furthermore, neural differentiation of ESCs was demonstrated in an experiment using poly(L-lactic acid) (PLLA) electrospun nanofibers incorporated with SWNTs and MWNTs. Scaffolds containing the carbon nanotubes promoted greater differentiation towards neural lineage, shown by an upregulation of Map-2. Differentiated cells aligned on the fibers demonstrating the influence of physical cues on cell morphology and lineage commitment [127]. Another recent study also investigated the effects of topography on human iPSC differentiation towards neuronal lineage [128]. A PDMS substrate was patterned with ridges/grooves of width 350 nm and groove depth of 300 nm, then single cells were placed on the nanostructures and allowed to differentiate for 4 days. Cell alignment on the 350 nm width groove substrate was compared to surfaces with 2 μm and 5 μm widths, and it was found that cells responded with the highest degree of alignment to the nanogrooves. Additionally, human iPSCs placed on the 350 nm substrate expressed the highest levels of neuroectodermal markers NPY and SYT4, demonstrating the importance of topography in guiding pluripotent stem cell phenotype. Collectively, these results indicate that controlling hPSC alignment on nanogroove structures directs cell differentiation towards neuronal lineage, and ESCs on random electrospun fibers incorporated with carbon nanotubes will purposefully elongate with the direction of the fibers and upregulate neuronal marker gene Map-2 (Figure 2).

MWNT films were employed to investigate the response of hESCs to surface roughness. hESC colonies favored rougher surfaces for attachment, exhibited fattened morphology with standard colony size, and retained pluripotency when cultured on MWNT films [129]. A similar study grafted CNTs with poly(acrylic acid) (PAA) to form a thin film, and the results indicated that this substrate, in combination with neural growth factors, stimulates hESC differentiation towards neural lineage at a higher rate than a conventional poly-L-ornithine (PLO) substrate often used in generating neurons from stem cells [130]. Another study utilized an array of CNTs conjugated with ECM proteins to determine the hPSC behavioral response when cultured on this platform. This array was found to support undifferentiated hESC and iPSC growth as well as self-renewal and pluripotency marker expression [131]. Furthermore, it was shown that both types of hPSCs cultured on the CNT arrays were able to differentiate towards ectoderm, mesoderm, and endoderm lineages [131]. The hPSCs grown on the CNT arrays were then directed towards spontaneous differentiation, and in reaction to the CNT topography, preferentially expressed mesodermal markers due to the physical stimuli exerted on the cells [131]. A similar study investigated culturing hESCs on a collagen/CNT matrix. Colonies were placed on tissue culture plates coated with gelatin, collagen, and collagen/CNTs and allowed to spontaneously differentiate. Colony morphology on the gelatin substrates was random and spread out, while hESCs on the collagen as well as the collagen/CNT matrices exhibited an elongated shape that aligned with the fibrils. By day 3, hESCs on the collagen/CNT surface expressed the early neural progenitor marker nestin significantly higher than the cells on the collagen substrate. By day 6, all three groups expressed nestin, with the highest levels detected in the collagen/CNT group followed by the collagen and gelatin groups, respectively [132]. These groundbreaking studies involving CNTs have provided insight on stimuli controlling hPSC lineage specification. CNT films maintain hPSC pluripotency and undifferentiated colony phenotype, substrates containing fibrillar architecture with CNTs promote hESC differentiation towards neural lineage, and CNT arrays exert physical forces on hPSCs that guide them towards mesoderm lineage commitment.

In another study, surface nanoroughness of silica-based glass wafters was altered and hESCs were placed on the various substrates in single cells. hESCs on the control glass surface demonstrated highly branched morphology with many cytoplasmic extensions, while cells on the nanorough glass were compact with few, short flapodia. Cells on a rough surface patterned with square-shaped smooth islands favored attachment to the smooth glass instead of the nanorough areas and expressed pluripotent marker Oct3/4, and hESCs placed on an exclusively rough surface spontaneously differentiated. Proliferation of hESC colonies was determined by placing cells on smooth glass and nanorough substrates, and it was determined that doubling time of hESCs on the control surface was 41 hours compared to a slower 71 hour doubling time of colonies on the rough surface [133]. In opposition, a study showed that silica colloidal crystal with diameters of 120, 400, and 600 nm coated with collagen I maintained the expression of murine ESC markers in comparison to smooth glass. However, colonies exhibited reduced spreading on the surface with altered topography [134]. Another study coated cell culture plates with poly[2-(methacryloyloxy)ethyl dimethyl-(3-sulfopropyl)ammonium hydroxide], then demonstrated that hESC colonies cultured on this substrate maintained their proliferation, self-renewal, and pluripotency capabilities [135]. A unique study used graphene and graphene oxide to coat glass coverslips and observed mouse iPSC behavior on the different substrates. It was found that iPSCs on the graphene and smooth glass surfaces proliferated at similar rates, but cells on the graphene oxide substrate had greater adhesion and proliferation. The graphene substrate maintained cells at an undifferentiated state, while the graphene oxide surface promoted spontaneous differentiation [136]. Overall, rough surfaces promote PSC adhesion with a more compact morphology, however, studies have found opposing evidence for whether or not nanorough surfaces maintain pluripotency and an undifferentiated state, or promote spontaneous differentiation. There are also conflicting results determining if these surfaces foster or hinder proliferation, therefore more studies are warranted to understand how PSCs respond to rough culture substrates.

## Conclusions

Tissue engineering has demonstrated the ability to generate desired cell types by combining the knowledge of biomaterials, stem cells, and signaling factors. The use of hPSCs in regenerative medicine has immense potential for treating numerous ailments, but experimental methods for solely and completely creating the desired tissues is necessary to avoid teratoma formation. Towards this goal, researchers have focused on mimicking *in vivo* microenvironmental cues in differentiation studies to parse which factors control lineage commitment. Trough carefully designed scaffolds and substrates, scientists have advanced the understanding of how nano-microenvironmental cues define cell behavior. By gaining this knowledge, stem cell differentiation can be further specified by combining nano-architecture and insoluble factors with other important biochemical cues.

## References

[1] Langer R, Vacanti JP (1993). Tissue engineering. Science.

[2] Zuk PA, Zhu M, Mizuno H, Huang J, Futrell JW (2001). Multilineage cells from human adipose tissue: implications for cell-based therapies. Tissue Eng.

[3] Zuk PA, Zhu M, Ashjian P, De Ugarte DA, Huang JI (2002). Human adipose tissue is a source of multipotent stem cells. Mol Biol Cell.

[4] Seo MJ, Suh SY, Bae YC, Jung JS (2005). Differentiation of human adipose stromal cells into hepatic lineage *in vitro* and *in vivo*. Biochem Biophys Res Commun.

[5] Cao Y, Sun Z, Liao L, Meng Y, Han Q (2005). Human adipose tissue-derived stem cells differentiate into endothelial cells *in vitro* and improve postnatal neovascularization *in vivo*. Biochem Biophys Res Commun.

[6] Kuznetsov SA, Krebsbach PH, Satomura K, Kerr J, Riminucci M (1997). Single-colony derived strains of human marrow stromal fibroblasts form bone after transplantation *in vivo*. J Bone Miner Res.

[7] Friedenstein AJ, Chailakhyan RK, Gerasimov UV (1987). Bone marrow osteogenic stem cells: *in vitro* cultivation and transplantation in diffusion chambers. Cell Tissue Kinet.

[8] Haynesworth SE, Goshima J, Goldberg VM, Caplan AI (1992). Characterization of cells with osteogenic potential from human marrow. Bone.

[9] Pittenger MF, Mackay AM, Beck SC, Jaiswal RK, Douglas R (1999). Multilineage potential of adult human mesenchymal stem cells. Science.

[10] Young HE, Black AC (2004). Adult stem cells. Anat Rec A Discov Mol Cell Evol Biol.

[11] Lin H (2002). The stem-cell niche theory: lessons from flies. Nat Rev Genet.

[12] Mauro A (1961). Satellite cell of skeletal muscle fibers. J Biophys Biochem Cytol.

[13] Cruess RL (1982). The Musculoskeletal system : embryology, biochemistry, and physiology.

[14] Owen M, Friedenstein AJ (1988). Stromal stem cells: marrow-derived osteogenic precursors. Ciba Found Symp.

[15] Gage FH, Coates PW, Palmer TD, Kuhn HG, Fisher LJ (1995). Survival and differentiation of adult neuronal progenitor cells transplanted to the adult brain. Proc Natl Acad Sci U S A.

[16] Janes SM, Lowell S, Hutter C (2002). Epidermal stem cells. J Pathol.

[17] Hayflick L (1965). The limited in vitro lifetime of human diploid cell strains. Exp Cell Res.

[18] Daley GQ, Scadden DT (2008). Prospects for stem cell-based therapy. Cell.

[19] Evans MJ, Kaufman MH (1981). Establishment in culture of pluripotential cells from mouse embryos. Nature.

[20] Martin GR (1981). Isolation of a pluripotent cell line from early mouse embryos cultured in medium conditioned by teratocarcinoma stem cells. Proc Natl Acad Sci U S A.

[21] Thomson JA, Itskovitz-Eldor J, Shapiro SS, Waknitz MA, Swiergiel JJ (1998). Embryonic stem cell lines derived from human blastocysts. Science.

[22] Xu C, Inokuma MS, Denham J, Golds K, Kundu P (2001). Feeder-free growth of undifferentiated human embryonic stem cells. Nat Biotechnol.

[23] Itskovitz-Eldor J, Schuldiner M, Karsenti D, Eden A, Yanuka O (2000). Differentiation of human embryonic stem cells into embryoid bodies compromising the three embryonic germ layers. Mol Med.

[24] Takahashi K, Yamanaka S (2006). Induction of pluripotent stem cells from mouse embryonic and adult fibroblast cultures by defined factors. Cell.

[25] Takahashi K, Tanabe K, Ohnuki M, Narita M, Ichisaka T (2007). Induction of pluripotent stem cells from adult human fibroblasts by defined factors. Cell.

[26] Yu J, Vodyanik MA, Smuga-Otto K, Antosiewicz-Bourget J, Frane JL (2007). Induced pluripotent stem cell lines derived from human somatic cells. Science.

[27] Tang C, Weissman IL, Drukker M (2012). The safety of embryonic stem cell therapy relies on teratoma removal. Oncotarget.

[28] Chang YL, Chen SJ, Kao CL, Hung SC, Ding DC (2012). Docosahexaenoic acid promotes dopaminergic differentiation in induced pluripotent stem cells and inhibits teratoma formation in rats with Parkinson-like pathology. Cell Transplant.

[29] Hashimoto J, Kariya Y, Miyazaki K (2006). Regulation of proliferation and chondrogenic differentiation of human mesenchymal stem cells by laminin-5 (laminin-332). Stem Cells.

[30] Mizuno M, Kuboki Y (2001). Osteoblast-related gene expression of bone marrow cells during the osteoblastic differentiation induced by type I collagen. J Biochem.

[31] Salasznyk RM, Williams WA, Boskey A, Batorsky A, Plopper GE (2004). Adhesion to Vitronectin and Collagen I Promotes Osteogenic Differentiation of Human Mesenchymal Stem Cells. J Biomed Biotechnol.

[32] Moursi AM, Globus RK, Damsky CH (1997). Interactions between integrin receptors and fibronectin are required for calvarial osteoblast differentiation *in vitro*. J Cell Sci.

[33] Discher DE, Mooney DJ, Zandstra PW (2009). Growth factors, matrices, and forces combine and control stem cells. Science.

[34] Authors Cohen DM, Chen CS (2008). Mechanical control of stem cell differentiation. Mechanical control of stem cell differentiation.

[35] Guilak F, Cohen DM, Estes BT, Gimble JM, Liedtke W (2009). Control of stem cell fate by physical interactions with the extracellular matrix. Cell Stem Cell.

[36] Wang JH, Thampatty BP (2008). Mechanobiology of adult and stem cells. Int Rev Cell Mol Biol.

[37] Sun Y, Chen CS, Fu J (2012). Forcing stem cells to behave: a biophysical perspective of the cellular microenvironment. Annu Rev Biophys.

[38] Lutolf MP, Hubbell JA (2005). Synthetic biomaterials as instructive extracellular microenvironments for morphogenesis in tissue engineering. Nat Biotechnol.

[39] Wu KC, Tseng CL, Wu CC, Kao FC, Tu YK (2013). Nanotechnology in the regulation of stem cell behavior. Sci Technol Adv Mat.

[40] Webster TJ, Stout DA (2012). Carbon nanotubes for stem cell control. Materials Today.

[41] Khademhosseini A, Langer R, Borenstein J, Vacanti JP (2006). Microscale technologies for tissue engineering and biology. Proc Natl Acad Sci U S A.

[42] Pirone DM, Chen CS (2004). Strategies for engineering the adhesive microenvironment. J Mammary Gland Biol Neoplasia.

[43] Yang F, Murugan R, Wang S, Ramakrishna S (2005). Electrospinning of nano/micro scale poly(L-lactic acid) aligned fibers and their potential in neural tissue engineering. Biomaterials.

[44] Kenawyel R, Layman JM, Watkins JR, Bowlin GL, Matthews JA (2003). Electrospinning of poly(ethylene-co-vinyl alcohol) fibers. Biomaterials.

[45] Pham QP, Sharma U, Mikos AG (2006). Electrospinning of polymeric nanofibers for tissue engineering applications: a review. Tissue Eng.

[46] Xu C, Inai R, Kotaki M, Ramakrishna S (2004). Electrospun nanofiber fabrication as synthetic extracellular matrix and its potential for vascular tissue engineering. Tissue Eng.

[47] Elias KL, Price RL, Webster TJ (2002). Enhanced functions of osteoblasts on nanometer diameter carbon fibers. Biomaterials.

[48] Zhang S (2003). Fabrication of novel biomaterials through molecular self-assembly. Nat Biotechnol.

[49] Menger FM (2002). Supramolecular chemistry and self-assembly. Proc Natl Acad Sci U S A.

[50] Estroff LA, Hamilton AD (2004). Water gelation by small organic molecules. Chem Rev.

[51] Xia YN, Whitesides GM (1998). Soft lithography. Angew Chem Int Edit.

[52] Ostuni E, Chen CS, Ingber DE, Whitesides GM (2001). Selective deposition of proteins and cells in arrays of microwells. Langmuir : the ACS journal of surfaces and colloids.

[53] Kane RS, Takayama S, Ostuni E, Ingber DE, Whitesides GM (1999). Patterning proteins and cells using soft lithography. Biomaterials.

[54] Ostuni E, Yan L, Whitesides GM (1999). The interaction of proteins and cells with self-assembled monolayers of alkanethiolates on gold and silver. Colloid Surface B.

[55] Xia YN, Whitesides GM (1998). Soft lithography. Annu Rev Mater Sci.

[56] Suh KY, Khademhosseini A, Yang JM, Eng G, Langer R (2004). Soft lithographic patterning of hyaluronic acid on hydrophilic substrates using molding and printing. Adv Mater.

[57] Khademhosseini A, Jon S, Suh KY, Tran TNT, Eng G (2003). Direct Patterning of protein- and cell-resistant polymeric monolayers and microstructures. Adv Mater.

[58] Harris GM, Shazly T, Jabbarzadeh E (2013). Deciphering the combinatorial roles of geometric, mechanical, and adhesion cues in regulation of cell spreading. PLoS One.

[59] Whitesides GM, Ostuni E, Takayama S, Jiang X, Ingber DE (2001). Soft lithography in biology and biochemistry. Annu Rev Biomed Eng.

[60] Brangwynne C, Huang S, Parker KK, Ingber DE, Ostuni E (2000). Symmetry breaking in cultured mammalian cells. In Vitro Cell Dev Biol Anim.

[61] Langer R, Tirrell DA (2004). Designing materials for biology and medicine. Nature.

[62] Peppas NA, Huang Y, Torres-Lugo M, Ward JH, Zhang J (2000). Physicochemical foundations and structural design of hydrogels in medicine and biology. Annu Rev Biomed Eng.

[63] Drury JL, Mooney DJ (2003). Hydrogels for tissue engineering: scaffold design variables and applications. Biomaterials.

[64] Hoffman AS (2002). Hydrogels for biomedical applications. Adv Drug Deliv Rev.

[65] Fisher OZ, Khademhosseini A, Langer R, Peppas NA (2010). Bioinspired materials for controlling stem cell fate. Acc Chem Res.

[66] Hutmacher DW (2001). Scaffold design and fabrication technologies for engineering tissues--state of the art and future perspectives. J Biomater Sci Polym Ed.

[67] Hoffman AS (2012). Hydrogels for biomedical applications. Adv Drug Deliver Rev.

[68] Zhang LJ, Webster TJ (2009). Nanotechnology and nanomaterials: Promises for improved tissue regeneration. Nano Today.

[69] Li X, Fan Y, Watari F (2010). Current investigations into carbon nanotubes for biomedical application. Biomed Mater.

[70] Kam NWS, Jessop TC, Wender PA, Dai HJ (2004). Nanotube molecular transporters: Internalization of carbon nanotube-protein conjugates into mammalian cells. Journal of the American Chemical Society.

[71] Klingeler R, Sim RB, SpringerLink (Online service) (2011). Carbon nanotubes for biomedical applications. Carbon nanostructures.

[72] Li X, Gao H, Uo M, Sato Y, Akasaka T (2009). Effect of carbon nanotubes on cellular functions *in vitro*. J Biomed Mater Res A.

[73] Cheng Q, Rutledge K, Jabbarzadeh E (2013). Carbon nanotube-poly(lactide-co-glycolide) composite scaffolds for bone tissue engineering applications. Ann Biomed Eng.

[74] Zanello LP, Zhao B, Hu H, Haddon RC (2006). Bone cell proliferation on carbon nanotubes. Nano Lett.

[75] Shi X, Sitharaman B, Pham QP, Liang F, Wu K (2007). Fabrication of porous ultra-short single-walled carbon nanotube nanocomposite scaffolds for bone tissue engineering. Biomaterials.

[76] Mikael PE, Amini AR, Basu J, Josefina Arellano-Jimenez M, Laurencin CT (2014). Functionalized carbon nanotube reinforced scaffolds for bone regenerative engineering: fabrication, *in vitro* and *in vivo* evaluation. Biomed Mater.

[77] Hu H, Ni Y, Montana V, Haddon RC, Parpura V (2004). Chemically Functionalized Carbon Nanotubes as Substrates for Neuronal Growth. Nano Lett.

[78] McKeon-Fischer KD, Flagg DH, Freeman JW (2011). Coaxial electrospun poly(Îμ-caprolactone), multiwalled carbon nanotubes, and polyacrylic acid/polyvinyl alcohol scaffold for skeletal muscle tissue engineering. J Biomed Mater Res A.

[79] Ni Y, Hu H, Malarkey EB, Zhao B, Montana V (2005). Chemically functionalized water soluble single-walled carbon nanotubes modulate neurite outgrowth. J Nanosci Nanotechnol.

[80] Whitesides GM (2006). The origins and the future of microfluidics. Nature.

[81] Lu H, Koo LY, Wang WM, Lauffenburger DA, Griffth LG (2004). Microfluidic shear devices for quantitative analysis of cell adhesion. Anal Chem.

[82] Takayama S, Ostuni E, LeDuc P, Naruse K, Ingber DE (2003). Selective chemical treatment of cellular microdomains using multiple laminar streams. Chem Biol.

[83] Glasgow IK, Zeringue HC, Beebe DJ, Choi SJ, Lyman JT (2001). Handling individual mammalian embryos using microfluidics. IEEE Trans Biomed Eng.

[84] Walters EM, Clark SG, Beebe DJ, Wheeler MB (2004). Mammalian embryo culture in a microfluidic device. Methods Mol Biol.

[85] Lucchetta EM, Lee JH, Fu LA, Patel NH, Ismagilov RF (2005). Dynamics of Drosophila embryonic patterning network perturbed in space and time using microfluidics. Nature.

[86] Chen CS (2008). Mechanotransduction - a field pulling together?. J Cell Sci.

[87] Geiger B, Spatz JP, Bershadsky AD (2009). Environmental sensing through focal adhesions. Nat Rev Mol Cell Biol.

[88] Osol G (1995). Mechanotransduction by vascular smooth muscle. J Vasc Res.

[89] Davies PF (1995). Flow-mediated endothelial mechanotransduction. Physiol Rev.

[90] Chien S (2007). Mechanotransduction and endothelial cell homeostasis: the wisdom of the cell. Am J Physiol Heart Circ Physiol.

[91] Engler AJ, Sen S, Sweeney HL, Discher DE (2006). Matrix elasticity directs stem cell lineage specification. Cell.

[92] Saha S, Ji L, de Pablo JJ, Palecek SP (2006). Inhibition of human embryonic stem cell differentiation by mechanical strain. J Cell Physiol.

[93] Saha S, Ji L, de Pablo JJ, Palecek SP (2008). TGFbeta/Activin/Nodal pathway in inhibition of human embryonic stem cell differentiation by mechanical strain. Biophys J.

[94] Chowdhury F, Na S, Li D, Poh YC, Tanaka TS (2010). Material properties of the cell dictate stress-induced spreading and differentiation in embryonic stem cells. Nat Mater.

[95] Keung AJ, Kumar S, Schaffer DV (2010). Presentation counts: microenvironmental regulation of stem cells by biophysical and material cues. Annu Rev Cell Dev Biol.

[96] Kim L, Vahey MD, Lee HY, Voldman J (2006). Microfluidic arrays for logarithmically perfused embryonic stem cell culture. Lab Chip.

[97] Illi B, Scopece A, Nanni S, Farsetti A, Morgante L (2005). Epigenetic histone modification and cardiovascular lineage programming in mouse embryonic stem cells exposed to laminar shear stress. Circ Res.

[98] Yamamoto K, Sokabe T, Watabe T, Miyazono K, Yamashita JK (2005). Fluid shear stress induces differentiation of Flk-1-positive embryonic stem cells into vascular endothelial cells in vitro. Am J Physiol Heart Circ Physiol.

[99] Adamo L, Naveiras O, Wenzel PL, McKinney-Freeman S, Mack PJ (2009). Biomechanical forces promote embryonic haematopoiesis. Nature.

[100] Chun TH, Hotary KB, Sabeh F, Saltiel AR, Allen ED (2006). A pericellular collagenase directs the 3-dimensional development of white adipose tissue. Cell.

[101] Young JL, Engler AJ (2011). Hydrogels with time-dependent material properties enhance cardiomyocyte differentiation *in vitro*. Biomaterials.

[102] Moore KA, Polte T, Huang S, Shi B, Alsberg E (2005). Control of basement membrane remodeling and epithelial branching morphogenesis in embryonic lung by Rho and cytoskeletal tension. Developmental dynamics: an official publication of the American Association of Anatomists.

[103] Chowdhury F, Li Y, Poh YC, Yokohama-Tamaki T, Wang N (2010). Soft substrates promote homogeneous self-renewal of embryonic stem cells via downregulating cell-matrix tractions. PLoS One.

[104] Folkman J, Moscona A (1978). Role of cell shape in growth control. Nature.

[105] McBeath R, Pirone DM, Nelson CM, Bhadriraju K, Chen CS (2004). Cell shape, cytoskeletal tension, and RhoA regulate stem cell lineage commitment. Dev Cell.

[106] Yang Y, Relan NK, Przywara DA, Schuger L (1999). Embryonic mesenchymal cells share the potential for smooth muscle differentiation: myogenesis is controlled by the cell's shape. Development.

[107] Manasek FJ, Burnside MB, Waterman RE (1972). Myocardial cell shape change as a mechanism of embryonic heart looping. Dev Biol.

[108] Ingber D (1991). Extracellular matrix and cell shape: potential control points for inhibition of angiogenesis. J Cell Biochem.

[109] Chen CS, Mrksich M, Huang S, Whitesides GM, Ingber DE (1997). Geometric control of cell life and death. Science.

[110] Bauwens CL, Peerani R, Niebruegge S, Woodhouse KA, Kumacheva E (2008). Control of human embryonic stem cell colony and aggregate size heterogeneity influences differentiation trajectories. Stem Cells.

[111] Peerani R, Rao BM, Bauwens C, Yin T, Wood GA (2007). Niche-mediated control of human embryonic stem cell self-renewal and differentiation. EMBO J.

[112] Stevens MM, George JH (2005). Exploring and engineering the cell surface interface. Science.

[113] Berkovitz BK, Pacy J (2002). Ultrastructure of the human intra-articular disc of the temporomandibular joint. Eur J Orthod.

[114] Dalby MJ, Riehle MO, Johnstone H, Affrossman S, Curtis ASG (2004). Investigating the limits of filopodial sensing: a brief report using SEM to image the interaction between 10 nm high nano-topography and fibroblast filopodia. Cell biology international.

[115] Wong JY, Leach JB, Brown XQ (2004). Balance of chemistry, topography, and mechanics at the cell-biomaterial interface: Issues and challenges for assessing the role of substrate mechanics on cell response. Surf Sci.

[116] Griffith LG, Swartz MA (2006). Capturing complex 3D tissue physiology *in vitro*. Nat Rev Mol Cell Biol.

[117] Curtis AS, Wilkinson CD (1998). Reactions of cells to topography. J Biomater Sci Polym Ed.

[118] Flemming RG, Murphy CJ, Abrams GA, Goodman SL, Nealey PF (1999). Effects of synthetic micro- and nano-structured surfaces on cell behavior. Biomaterials.

[119] Lim JY, Donahue HJ (2007). Cell sensing and response to micro- and nanostructured surfaces produced by chemical and topographic patterning.

[120] Kong HJ, Mooney DJ (2007). Microenvironmental regulation of biomacromolecular therapies. Nat Rev Drug Discov.

[121] Reilly GC, Engler AJ (2010). Intrinsic extracellular matrix properties regulate stem cell differentiation. S J Biomech.

[122] Chen A, Lieu DK, Freschauf L, Lew V, Sharma H (2011). Shrink-film configurable multiscale wrinkles for functional alignment of human embryonic stem cells and their cardiac derivatives. Adv Mater.

[123] Liu L, Yuan Q, Shi J, Li X, Jung D (2012). Chemically-defined scaffolds created with electrospun synthetic nanofibers to maintain mouse embryonic stem cell culture under feeder-free conditions. Biotechnol Lett.

[124] Gerecht S, Bettinger CJ, Zhang Z, Borenstein JT, Vunjak-Novakovic G (2007). The effect of actin disrupting agents on contact guidance of human embryonic stem cells. Biomaterials.

[125] Nur-E-Kamal A, Ahmed I, Kamal J, Babu AN, Schindler M (2008). Covalently attached FGF-2 to three-dimensional polyamide nanofibrillar surfaces demonstrates enhanced biological stability and activity. Mol Cell Biochem.

[126] Lee MR, Kwon KW, Jung H, Kim HN, Suh KY (2010). Direct differentiation of human embryonic stem cells into selective neurons on nanoscale ridge/groove pattern arrays. Biomaterials.

[127] Kabiri M, Soleimani M, Shabani I, Futrega K, Ghaemi N (2012). Neural differentiation of mouse embryonic stem cells on conductive nanofiber scaffolds. Biotechnol Lett.

[128] Pan F, Zhang M, Wu G, Lai Y, Greber B (2013). Topographic effect on human induced pluripotent stem cells differentiation towards neuronal lineage. Biomaterials.

[129] Brunner EW, Jurewicz I, Heister E, Fahimi A, Bo C (2014). Growth and proliferation of human embryonic stem cells on fully synthetic scaffolds based on carbon nanotubes. ACS Appl Mater Interfaces.

[130] Chao TI, Xiang S, Chen CS, Chin WC, Nelson AJ (2009). Carbon nanotubes promote neuron differentiation from human embryonic stem cells. Biochem Biophys Res Commun.

[131] Pryzhkova MV, Aria I, Cheng Q, Harris GM, Zan X (2014). Carbon nanotube-based substrates for modulation of human pluripotent stem cell fate. Biomaterials.

[132] Sridharan I, Kim T, Wang R (2009). Adapting collagen/CNT matrix in directing hESC differentiation. Biochem Biophys Res Commun.

[133] Chen W, Villa-Diaz LG, Sun Y, Weng S, Kim JK (2012). Nanotopography influences adhesion, spreading, and self-renewal of human embryonic stem cells. ACS Nano.

[134] Ji L, LaPointe VL, Evans ND, Stevens MM (2012). Changes in embryonic stem cell colony morphology and early differentiation markers driven by colloidal crystal topographical cues. Eur Cell Mater.

[135] Villa-Diaz LG, Nandivada H, Ding J, Nogueira-de-Souza NC, Krebsbach PH (2010). Synthetic polymer coatings for long-term growth of human embryonic stem cells. Nat Biotechnol.

[136] Chen GY, Pang DW, Hwang SM, Tuan HY, Hu YC (2012). A graphene-based platform for induced pluripotent stem cells culture and differentiation. Biomaterials.

